# Mini-Open Fifth Metatarsal Osteotomy with Intramedullary Rigid Fixation for Symptomatic Coughlin Type II and III Bunionette Deformity

**DOI:** 10.3390/jcm15135134

**Published:** 2026-07-01

**Authors:** Mesut Uluöz, Mehmet Yiğit Gökmen, Özhan Pazarcı, Evren Karaali, Osman Çiloğlu

**Affiliations:** 1 Department of Orthopaedics and Traumatology, Adana City Training and Research Hospital, University of Health Sciences, Adana 01230, Türkiye; dr.pazarci@gmail.com (Ö.P.); drevrenkaraali@gmail.com (E.K.); osmanciloglu@gmail.com (O.Ç.); 2 Department of Orthopaedics and Traumatology, Faculty of Medicine, Çanakkale Onsekiz Mart University, Çanakkale 17110, Türkiye; mehmet_yigit_gokmen@hotmail.com

**Keywords:** tailor’s bunion, bunionette, osteotomy, orthopedic procedures, intramedullary plating, miniplate

## Abstract

**Background**: Bunionette deformity is commonly treated with distal or diaphyseal osteotomy, but concerns remain regarding correction loss, implant irritation, and metatarsal shortening. This study evaluated outcomes of mini-open fifth metatarsal osteotomy stabilized with intramedullary rigid fixation in symptomatic Coughlin type II and III deformity. **Methods**: This single-center retrospective observational study included 32 consecutive patients treated between February 2018 and February 2023. Radiographic outcomes included the fourth-to-fifth intermetatarsal angle (IMA), fifth metatarsophalangeal angle (MPA), maintenance of correction, and fifth metatarsal shortening. Clinical outcomes included the American Orthopaedic Foot and Ankle Society (AOFAS) score, visual analog scale (VAS) pain score, and complications. An exploratory subgroup analysis compared isolated correction with combined procedures. **Results**: The mean follow-up was 31.5 ± 6.8 months. The mean AOFAS score improved from 52.5 ± 4.2 to 93.4 ± 3.4, and the mean VAS score decreased from 7.8 ± 0.9 to 1.2 ± 0.6 (both *p* < 0.001). The mean MPA improved from 19.4° ± 3.6° to 2.3° ± 1.1°, and the mean IMA improved from 14.0° ± 1.4° to 4.5° ± 2.5° (both *p* < 0.001). Minor but statistically significant correction loss occurred between early postoperative and final follow-up radiographs. Mean fifth metatarsal shortening was 1.3 ± 0.8 mm. One patient required implant removal for hardware irritation. No nonunion, transfer metatarsalgia, or wound complications were observed. **Conclusions**: Mini-open fifth metatarsal osteotomy with intramedullary rigid fixation was associated with pain relief, functional improvement, maintained radiographic correction, limited shortening, and a low observed complication rate in this series.

## 1. Introduction

Bunionette deformity, also known as tailor’s bunion, is a painful prominence of the lateral aspect of the fifth metatarsal head that may cause local inflammation, keratosis, and difficulty with footwear [1,2]. Although many patients improve with shoe modification, padding, and other conservative measures, surgery is generally reserved for patients with persistent symptoms and functional limitations despite nonoperative treatment [3].

Radiographic assessment plays a central role in treatment planning. The Coughlin classification remains widely used because it links deformity morphology to surgical decision-making by distinguishing an enlarged fifth metatarsal head from deformities caused by lateral bowing of the fifth metatarsal or widening of the fourth-fifth intermetatarsal angle [4]. In symptomatic patients, type III deformity appears to be the most common pattern, which supports the use of osteotomy-based correction rather than isolated exostectomy in deformities driven by angular malalignment [5,6].

Many operative techniques have been described for bunionette correction, including exostectomy and distal, diaphyseal, or proximal osteotomies performed through open, mini-open, or percutaneous approaches [7]. However, no single technique has emerged as clearly superior. Meta-analytic evidence shows that all osteotomy groups improve radiographic alignment, with greater angular correction after proximal osteotomies but fewer major complications after distal procedures [8]. Systematic review data on minimally invasive and percutaneous surgery also support improvement in radiographic and patient-reported outcomes, while highlighting major heterogeneity in surgical technique, fixation method, and postoperative management, as well as the predominance of retrospective case series [9]. International consensus statements likewise support osteotomy-based correction but reflect ongoing variation in osteotomy level, fixation strategy, and perioperative protocol [10].

These uncertainties are especially relevant in Coughlin type II and III deformities, where treatment must address both angular correction and construct stability. Percutaneous techniques without internal fixation have shown encouraging results, but concerns remain regarding maintenance of correction, metatarsal shortening, secondary callus formation, and sagittal-plane malalignment [11]. Open diaphyseal and oblique osteotomies can also achieve effective correction, but they often require wider exposure or conventional implants, which may be less well tolerated in the thin soft-tissue envelope of the fifth metatarsal [5,12].

Fixation strategies that preserve soft tissue while providing stable internal support remain attractive. We adapted a mini-open intramedullary low-profile miniplate technique, previously used in forefoot reconstruction, for bunionette correction. The aim of this study was to evaluate the radiographic and clinical outcomes of mini-open fifth metatarsal osteotomy with intramedullary rigid fixation in symptomatic Coughlin type II and III bunionette deformities, focusing on maintenance of correction, metatarsal shortening, and complications, rather than on a direct comparison with other surgical techniques.

## 2. Materials and Methods

### 2.1. Study Design, Setting, and Participants

This single-center retrospective observational study evaluated radiographic and clinical outcomes after mini-open fifth metatarsal osteotomy stabilized with intramedullary rigid fixation for bunionette deformity. The study was conducted at Adana City Training and Research Hospital, a tertiary referral center for foot and ankle surgery.

All consecutive patients who underwent surgical correction of bunionette deformity using the described technique between February 2018 and February 2023 were screened for eligibility. Inclusion criteria were age 18 years or older; diagnosis of symptomatic Coughlin type II or III bunionette deformity; failure of at least 6 months of conservative treatment; availability of standardized weight-bearing foot radiographs (anteroposterior and lateral) obtained preoperatively, in the early postoperative period, and at final follow-up; and a minimum clinical follow-up of 12 months.

Patients were excluded if they had uncontrolled diabetes mellitus, post-traumatic deformity of the fifth ray, inflammatory arthropathy, neuromuscular disease, prior surgery involving the fifth metatarsal, incomplete radiographic documentation, or loss to follow-up before radiographic union.

Concomitant hallux valgus correction performed during the same operative session was recorded. Outcomes were analyzed for the overall cohort, and subgroup analyses were performed comparing isolated bunionette surgery with combined procedures to address potential confounding related to simultaneous medial column correction. These subgroup analyses were considered exploratory due to the limited number of patients in each subgroup, and no equivalence or non-inferiority inferences were drawn.

### 2.2. Surgical Technique

All procedures were performed by a single foot-and-ankle specialist surgeon using a standardized protocol. Under spinal anesthesia, a mini-open lateral incision was made over the planned osteotomy level, which was determined under fluoroscopic guidance according to the deformity location. In Coughlin type II deformities, the osteotomy was planned at the level of the lateral bowing of the fifth metatarsal to allow correction of the curved metatarsal axis. In Coughlin type III deformities, the osteotomy was placed more proximally at the distal diaphyseal/metaphyseal region to permit medial translation and angular correction of the widened fourth-fifth intermetatarsal angle. A low-energy transverse osteotomy was created using multiple drill holes followed by completion with a narrow osteotome. In this study, “low-energy osteotomy” refers to an osteotomy performed by sequential drill-hole weakening and manual completion with an osteotome, without the use of an oscillating saw, in order to minimize thermal injury and uncontrolled bone loss. An oscillating saw was not used.

After osteotomy, the distal fragment was translated medially using a curved clamp placed within the proximal fragment. When required, additional angular correction was achieved under fluoroscopic control. Once satisfactory correction was confirmed, rigid fixation was performed with an intramedullary TRUE LOCK 2.0 mm Mini Plate system (TRUEMED Medikal Ürünler Üretim ve Pazarlama A.Ş., İstanbul, Türkiye), manufactured from Ti6Al4V ELI material according to ASTM F136 standards. The proximal portion of the plate was seated intramedullary within the proximal fragment, while the distal portion was secured to the distal fragment with two locking screws from the same 2.0 mm system. Final alignment and hardware position were confirmed fluoroscopically. The wound was closed in layers, and no drain was routinely used.

### 2.3. Postoperative Management and Follow-Up Schedule

No cast or splint immobilization was applied. Patients were allowed early protected ambulation using a postoperative shoe, with heel weight-bearing as tolerated. Clinical follow-up was scheduled at approximately 3 weeks for wound evaluation and suture removal, and radiographic follow-up was routinely obtained at 6 and 12 weeks postoperatively, with additional visits as clinically indicated. Full weight-bearing was typically initiated at 6 weeks based on clinical assessment and radiographic progression.

### 2.4. Radiographic Evaluation

Standardized weight-bearing anteroposterior and lateral foot radiographs were assessed at three time points: preoperative; early postoperative (defined as the first postoperative weight-bearing radiograph obtained within 6 weeks after surgery); and final follow-up (last available weight-bearing radiograph).

Radiographic measurements were performed using a standardized method. The fourth-to-fifth intermetatarsal angle (IMA) was measured on weight-bearing anteroposterior radiographs as the angle between the longitudinal axes of the fourth and fifth metatarsals. The fifth metatarsophalangeal angle (MPA) was measured on anteroposterior radiographs as the angle between the longitudinal axis of the fifth metatarsal and the longitudinal axis of the proximal phalanx of the fifth toe.

Maintenance of correction was evaluated by comparing early postoperative and final follow-up measurements. Loss of correction was expressed as the change over time (final minus early postoperative) for IMA and MPA.

Fifth metatarsal shortening was quantified as the change in fifth metatarsal length (in millimeters) between early postoperative and final follow-up anteroposterior weight-bearing radiographs, measured using the institutional Picture Archiving and Communication System (PACS) calibration tools after digital calibration of the radiographs.

Radiographic measurements were performed using the institutional PACS system according to a standardized measurement protocol. All measurements were independently performed by two orthopedic surgeons who were blinded to the clinical outcome scores at the time of measurement. The mean value of the two measurements was used for analysis. Formal interobserver and intraobserver reliability analyses were not performed.

### 2.5. Clinical Outcomes and Complications

Pain was assessed using a visual analog scale (VAS) preoperatively and at the final follow-up visit. Functional outcome was assessed using the American Orthopaedic Foot and Ankle Society (AOFAS) score preoperatively and at final follow-up. Medical records were reviewed for postoperative complications, including wound problems, symptomatic lateral forefoot hyperkeratosis, hardware irritation, implant removal, delayed union, and nonunion.

Radiographic union was defined as progressive bridging callus across the osteotomy site with resolution of the osteotomy line on serial radiographs, in conjunction with clinical recovery as documented in follow-up records. Delayed union was defined as a lack of progressive radiographic healing by 12 weeks, and nonunion was defined as the absence of union by 6 months.

### 2.6. Statistical Analysis

Continuous variables were reported as mean ± standard deviation. Categorical variables were reported as counts and percentages. Normality was assessed using the Shapiro–Wilk test. Comparisons between preoperative and final follow-up values and between early postoperative and final follow-up radiographic measurements were performed using the Wilcoxon signed-rank test. Between-group comparisons in the subgroup analysis were performed using the Mann–Whitney U test. Because fifth metatarsal shortening was analyzed as a single change variable, it was summarized descriptively and was not included in the longitudinal paired comparison analysis. Subgroup analyses were considered exploratory because of the limited number of patients in each subgroup, and no equivalence or non-inferiority inference was made. A two-sided *p*-value below 0.05 was considered statistically significant. Analyses were performed using IBM SPSS Statistics for Windows, Version 23.0 (IBM Corp., Armonk, NY, USA).

## 3. Results

### 3.1. Demographics and Baseline Characteristics

A total of 32 patients (25 females, 7 males) were included. The mean age was 46.4 ± 11.4 years (range, 22–66). Surgery was performed on 15 right and 17 left feet. According to the Coughlin classification, 14 patients (43.8%) had type II and 18 (56.2%) had type III bunionette deformity. All patients had failed at least 6 months of conservative management prior to surgery. The mean clinical follow-up was 31.5 ± 6.8 months. Baseline characteristics are summarized in Table 1.

### 3.2. Clinical and Functional Outcomes

Clinical outcome measures improved significantly from baseline to final follow-up (Table 2). The mean AOFAS score increased from 52.5 ± 4.2 preoperatively to 93.4 ± 3.4 at final follow-up (*p* < 0.001). The mean VAS pain score decreased from 7.8 ± 0.9 preoperatively to 1.2 ± 0.6 at final follow-up (*p* < 0.001). No cases of persistent or worsening pain were recorded.

### 3.3. Radiographic Evaluation and Correction Maintenance

**Figure 1:**
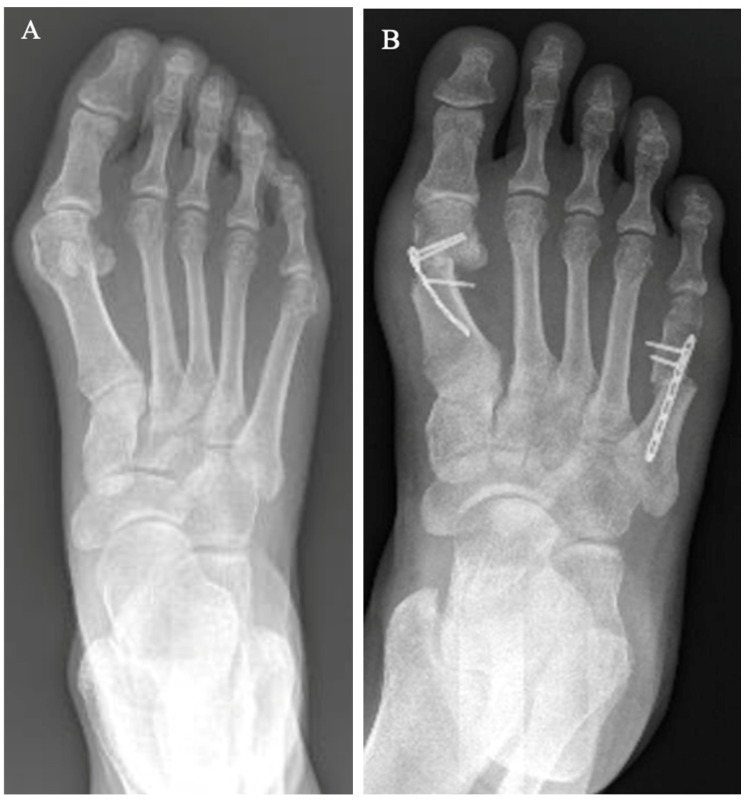
Weight-bearing anteroposterior radiographs of a 45-year-old female patient with symptomatic Coughlin type III bunionette deformity. (**A**) Preoperative radiograph showing lateral prominence of the fifth metatarsal head and increased fifth metatarsophalangeal and fourth-fifth intermetatarsal angles. (**B**) Postoperative radiograph obtained after mini-open fifth metatarsal osteotomy and intramedullary TRUE LOCK 2.0 mm Mini Plate fixation, demonstrating medial translation of the distal fifth metatarsal fragment, improved fifth ray alignment, and stable implant position.

Radiographic assessment demonstrated significant correction at final follow-up (Table 2). The mean fifth metatarsophalangeal angle (MPA) improved from 19.4° ± 3.6° preoperatively to 2.3° ± 1.1° at final follow-up (*p* < 0.001), and the mean fourth–fifth intermetatarsal angle (IMA) decreased from 14.0° ± 1.4° to 4.5° ± 2.5° (*p* < 0.001). Representative radiographs demonstrating the preoperative deformity and postoperative correction are shown in Figure 1.

Between the early postoperative assessment and final follow-up, small but statistically significant increases were observed in both MPA and IMA, indicating minor loss of correction over time (Table 3). Mean fifth metatarsal shortening was 1.3 ± 0.8 mm, with no evidence of nonunion or transfer metatarsalgia.

### 3.4. Complications

Hardware irritation requiring implant removal occurred in one patient. No cases of superficial infection, delayed wound healing, symptomatic lateral forefoot hyperkeratosis, delayed union, nonunion, or transfer metatarsalgia were observed during follow-up.

### 3.5. Subgroup Analysis: Isolated Versus Combined Procedures

Nineteen patients (59.4%) underwent isolated bunionette correction, and 13 (40.6%) underwent combined procedures. In this exploratory subgroup analysis, no statistically significant between-group differences were detected in final clinical or radiographic outcomes. Final AOFAS scores were 94.0 ± 3.5 in the isolated group and 92.8 ± 3.3 in the combined group (*p* = 0.326), while final VAS pain scores were 1.1 ± 0.7 and 1.2 ± 0.4, respectively (*p* = 0.496). No statistically significant difference in IMA correction was detected between groups (*p* = 0.748). Given the limited number of patients in each subgroup, these findings should be interpreted as descriptive and exploratory observations rather than evidence of equivalence between isolated and combined procedures.

## 4. Discussion

The principal finding of this study is that mini-open fifth metatarsal osteotomy stabilized with intramedullary low-profile miniplate fixation was associated with marked clinical improvement and generally maintained radiographic correction in patients with Coughlin type II–III bunionette deformity. Although a small but statistically significant increase in MPA and IMA occurred between the early postoperative assessment and final follow-up, the magnitude of change was limited, supporting overall maintenance of correction within the follow-up period of this retrospective series.

A variety of operative strategies have been described for bunionette deformity, including lateral metatarsal head procedures and osteotomy-based correction. Earlier approaches, such as metatarsal head resection or lateral condylar resection, may provide symptom relief, yet concerns regarding joint instability, recurrence, and long-term degenerative changes have been reported, which have contributed to the broader transition toward osteotomy-based correction in contemporary practice [13,14]. This morphology-based approach is also supported by review literature, which emphasizes selecting the osteotomy level according to the underlying deformity pattern rather than using a single procedure for all bunionette types [15].

For Coughlin type II–III deformities, correction is conceptually driven by the intermetatarsal divergence and the resulting increase in the fifth metatarsophalangeal angle, making osteotomy-based strategies a commonly used treatment approach. In this context, diaphyseal oblique osteotomies based on translation and rotational principles (e.g., Mau-type and Ludloff-type) have demonstrated favorable correction and clinical outcomes; however, these methods typically require greater exposure and conventional fixation, and implant-related symptoms may necessitate removal in some cases [5]. In contrast, the present technique positions the fixation intramedullary to provide stable internal support while reducing subcutaneous implant prominence. An additional practical advantage observed in our protocol is that it allows early protected ambulation without cast immobilization.

Techniques adapted from hallux valgus surgery have also been applied to bunionette deformity. Distal chevron osteotomy with absorbable pin fixation has been reported as another fixation-based option for symptomatic bunionette deformity [16]. Reverse-scarf osteotomy with screw fixation has shown promising short-term outcomes but generally requires wider exposure and saw-based osteotomy creation [12]. Alternative implant-free adaptations, such as fifth metatarsal scarf osteotomy stabilized without hardware, have been described but may be technically demanding and can be associated with secondary procedures in the setting of symptoms such as transfer metatarsalgia [17]. In the current series, mean fifth metatarsal shortening was limited (1.3 mm), and no transfer metatarsalgia was documented. These findings suggest that satisfactory alignment can be achieved with this technique in selected patients; however, forefoot biomechanics and plantar pressure distribution were not directly assessed in the present study.

Minimally invasive and percutaneous techniques have expanded substantially with the increased availability of burr systems. Percutaneous distal osteotomy stabilized with a Kirschner wire can yield good correction but requires prolonged external fixation, which may increase pin-related risks and temporarily restrict metatarsophalangeal motion [18]. Implant-free percutaneous distal oblique osteotomies have also shown improvement in pain and alignment, but metatarsal shortening and secondary callus-related symptoms such as hyperkeratosis have been described in some series [19,20,21]. More recent minimally invasive series, including sliding distal metatarsal minimally invasive osteotomy and minimally invasive distal chevron osteotomy without fixation, have also reported favorable clinical and radiographic outcomes, but they remain heterogeneous in terms of osteotomy geometry, fixation strategy, postoperative protocol, and reported radiographic parameters [22,23]. These issues are clinically relevant because symptomatic hyperkeratotic callus may affect shoe wear during the remodeling period. In our cohort, symptomatic hyperkeratosis was not observed; however, whether intramedullary fixation reduces secondary callus-related symptoms compared with other techniques cannot be determined without a control group.

Vascular complications represent another consideration. A survey-based consensus on percutaneous bunionette correction emphasized technique standardization and highlighted preferences toward percutaneous oblique osteotomy; however, broader concerns remain regarding iatrogenic soft-tissue injury and vascular compromise, particularly with more proximal open exposures [10]. In the present cohort, no osteonecrosis was observed, which may relate to the limited dissection afforded by the mini-open approach, though definitive conclusions cannot be drawn from a single series.

Recent evidence syntheses have attempted to compare correction and complication profiles across osteotomy levels and techniques. A meta-analysis focusing on fifth metatarsal osteotomies reported that distal osteotomies tend to have fewer major complications, whereas proximal osteotomies can achieve larger angular corrections [8]. Similarly, a contemporary systematic review of minimally invasive and percutaneous bunionette surgery suggested overall improvements in radiographic alignment and patient-reported outcomes, although heterogeneity in techniques and fixation strategies limits direct comparisons [9]. In our practice, osteotomy level was individualized according to deformity severity, with a more proximal osteotomy generally preferred for Coughlin type III deformity. The present technique should therefore be interpreted as a technical option that combines limited exposure with internal fixation, rather than as evidence of superiority over minimally invasive or traditional open techniques.

Finally, concomitant procedures are a potential confounder when interpreting clinical improvement. In our exploratory subgroup analysis, no statistically significant differences were detected in final functional outcomes, pain scores, or IMA correction between isolated bunionette correction and combined procedures. Because the subgroup sample sizes were small, these findings should not be interpreted as evidence of equivalence between isolated and combined procedures, but only as descriptive observations within this cohort.

### Limitations

This study has several limitations. Its retrospective single-center design and relatively small sample size may limit the generalizability of the findings, particularly for subgroup analyses. All procedures were performed by a single experienced foot-and-ankle surgeon, which ensured technical consistency but may also limit external validity. The absence of a control group prevents direct comparison with other established bunionette correction techniques. Radiographic measurements were obtained using a standardized protocol; however, formal interobserver and intraobserver reliability analyses were not performed. In addition, the AOFAS score was used as the primary functional outcome measure, although it has recognized limitations compared with contemporary patient-reported outcome instruments. Although no major complications were observed, this finding should be interpreted cautiously because uncommon adverse events may not be detected in a limited cohort. Longer-term comparative studies with larger samples are needed to confirm these findings.

## 5. Conclusions

Mini-open fifth metatarsal osteotomy with intramedullary rigid fixation was associated with favorable clinical and radiographic outcomes in patients with symptomatic Coughlin type II and III bunionette deformity. In this series, the technique was associated with marked improvement in pain and function, substantial correction of the deformity, limited metatarsal shortening, and no observed nonunion or transfer metatarsalgia. Although a small degree of radiographic correction loss occurred during follow-up, the overall maintenance of alignment remained satisfactory. These findings support this technique as a feasible surgical option in appropriately selected patients; however, comparative studies with larger cohorts are needed to determine its relative advantages over other bunionette correction methods.

## Figures and Tables

**Table 1:** Patient demographics and baseline characteristics.

Characteristic	Value (*n* = 32)
Age, years, mean ± SD (range)	46.4 ± 11.4 (22–66)
Sex, female/male, *n* (%)	25 (78.1)/7 (21.9)
Affected side, right/left, *n*	15/17
Clinical follow-up, months, mean ± SD	31.5 ± 6.8
Coughlin classification, type II/type III, *n* (%)	14 (43.8)/18 (56.2)
Prior conservative treatment, months, mean ± SD	8.4 ± 2.1

Values are presented as mean ± standard deviation unless otherwise stated. Percentages were calculated based on the total study population (*n* = 32). Coughlin classification refers to the radiographic severity of bunionette deformity. SD, standard deviation.

**Table 2:** Clinical and radiographic outcomes before surgery and at final follow-up.

Parameter	Preoperative	Final Follow-Up	*p*-Value ^†^
AOFAS score	52.5 ± 4.2	93.4 ± 3.4	<0.001
VAS pain score	7.8 ± 0.9	1.2 ± 0.6	<0.001
Fifth metatarsophalangeal angle (MPA), °	19.4 ± 3.6	2.3 ± 1.1	<0.001
Fourth–fifth intermetatarsal angle (IMA), °	14.0 ± 1.4	4.5 ± 2.5	<0.001

Values are presented as mean ± standard deviation. ^†^ Comparisons between preoperative and final follow-up values were performed using the Wilcoxon signed-rank test. A two-sided *p*-value < 0.05 was considered statistically significant. AOFAS, American Orthopaedic Foot and Ankle Society; VAS, visual analog scale; MPA, metatarsophalangeal angle; IMA, intermetatarsal angle.

**Table 3:** Radiographic stability from early postoperative assessment to final follow-up.

Radiographic Parameter	Early Postoperative, Mean ± SD	Final Follow-Up, Mean ± SD	*p*-Value ^†^
Fifth metatarsophalangeal angle (MPA), °	0.8 ± 0.7	2.3 ± 1.1	0.002
Fourth–fifth intermetatarsal angle (IMA), °	3.1 ± 1.2	4.5 ± 2.5	0.004

Values are presented as mean ± standard deviation. ^†^ Comparisons between early postoperative and final follow-up measurements were performed using the Wilcoxon signed-rank test. A two-sided *p*-value < 0.05 was considered statistically significant. SD, standard deviation; MPA, metatarsophalangeal angle; IMA, intermetatarsal angle.

## Data Availability

The data presented in this study are available on reasonable request from the corresponding author. The data are not publicly available because they contain information that could compromise patient privacy and are subject to institutional data protection policies.

## Abbreviations

AOFAS	American Orthopaedic Foot and Ankle Society score
IMA	Fourth-to-fifth intermetatarsal angle
MPA	Fifth metatarsophalangeal angle
PACS	Picture Archiving and Communication System
SD	Standard deviation
SPSS	Statistical Package for the Social Sciences
VAS	Visual analog scale
